# Hair Growth Promoting Effects of 15-Hydroxyprostaglandin Dehydrogenase Inhibitor in Human Follicle Dermal Papilla Cells

**DOI:** 10.3390/ijms25137485

**Published:** 2024-07-08

**Authors:** Hye Won Lim, Hak Joong Kim, Chae Young Jeon, Yurim Lee, Mujun Kim, Jinsick Kim, Soon Re Kim, Sanghwa Lee, Dong Chul Lim, Hee Dong Park, Byung Cheol Park, Dong Wook Shin

**Affiliations:** 1 Research Institute for Biomedical and Health Science, Konkuk University, Chungju 27478, Chungcheongbuk-do, Republic of Korea; hyewon0225@kku.ac.kr (H.W.L.); young4mam@kku.ac.kr (C.Y.J.); besy100@kku.ac.kr (M.K.); jindoli477@kku.ac.kr (J.K.); 2 Innovo Therapeutics Inc., 507, Mapo-daero 38, Mapo-gu, Seoul 04174, Republic of Korea; hakkim@innovothera.com (H.J.K.); yrlee@innovothera.com (Y.L.); shlee@innovothera.com (S.L.); dclim@innovothera.com (D.C.L.); hdpark@innovothera.com (H.D.P.); 3 Basic and Clinical Hair Institute, Dankook University, 201, Manghyang-ro, Dongnam-gu, Cheonan-si 31116, Chungcheongnam-do, Republic of Korea; sl715@nate.com (S.R.K.); 4exodus@dankook.ac.kr (B.C.P.); 4 Department of Dermatology, Dankook University Hospital, 201, Manghyang-ro, Dongnam-gu, Cheonan-si 31116, Chungcheongnam-do, Republic of Korea

**Keywords:** 15-prostaglandin dehydrogenase inhibitor, hair growth, human follicle dermal papilla cells, dihydrotestosterone

## Abstract

Prostaglandin E_2_ (PGE_2_) is known to be effective in regenerating tissues, and bimatoprost, an analog of PGF_2α_, has been approved by the FDA as an eyelash growth promoter and has been proven effective in human hair follicles. Thus, to enhance PGE_2_ levels while improving hair loss, we found dihydroisoquinolinone piperidinylcarboxy pyrazolopyridine (DPP), an inhibitor of 15-hydroxyprostaglandin dehydrogenase (15-PGDH), using DeepZema^®^, an AI-based drug development program. Here, we investigated whether DPP improved hair loss in human follicle dermal papilla cells (HFDPCs) damaged by dihydrotestosterone (DHT), which causes hair loss. We found that DPP enhanced wound healing and the expression level of alkaline phosphatase in DHT-damaged HFDPCs. We observed that DPP significantly down-regulated the generation of reactive oxygen species caused by DHT. DPP recovered the mitochondrial membrane potential in DHT-damaged HFDPCs. We demonstrated that DPP significantly increased the phosphorylation levels of the AKT/ERK and activated Wnt signaling pathways in DHT-damaged HFDPCs. We also revealed that DPP significantly enhanced the size of the three-dimensional spheroid in DHT-damaged HFDPCs and increased hair growth in ex vivo human hair follicle organ culture. These data suggest that DPP exhibits beneficial effects on DHT-damaged HFDPCs and can be utilized as a promising agent for improving hair loss.

## 1. Introduction

Hair loss is a prevalent occurrence among individuals of varying ages [1]. Given the significant association between hair and cultural identity, the onset of hair loss can profoundly impact one’s overall well-being and quality of life [2,3]. Androgenetic alopecia (AGA) is the predominant hair loss condition among men, characterized by the gradual replacement of thick terminal hair with fine vellus hair in genetically predisposed areas of the scalp, notably the frontal and vertex regions [4]. The principal pathological changes observed in AGA involve alterations in hair follicle dynamics, marked by a progressive shortening of the anagen phase. The pivotal role of 5-reductase in dermal papilla cells is central to these changes, facilitating the conversion of testosterone (T) into DHT. Subsequently, strong binding of DHT to androgen receptors (AR) triggers a cascade of signaling events that disrupt normal hair growth, ultimately leading to the miniaturization of affected hair follicles [5,6]. Despite these insights, the precise mechanisms underlying AGA remain incompletely elucidated.

HFDPCs are a mesenchymal type of cell that connects to capillaries under the hair follicle [7,8]. They have also been found to possess stem cell capabilities and are expected to play an important role in preventing hair loss and hair growth. Cell division and migration around the dermal papilla are closely related to hair growth. During the growth phase, new hair is created from the dermal papilla. Cells are activated by various cytokines and hormones, resulting in cell migration to the dermal papilla and affecting hair growth [7,8,9].

Numerous studies have indicated a correlation between the expression levels of prostaglandins (PGs) and hair growth [10,11,12]. Prostanoids, such as PGs and thromboxane A2 (TXA2), constitute a lipid-derived group of autacoids that influence numerous physiological systems and pathological conditions [13,14]. They are produced through the sequential metabolism of arachidonic acid by cyclooxygenase, resulting in the formation of PGH_2_, which is subsequently transformed into Prostaglandin D2 (PGD_2_), prostaglandin I2 (PGI_2_), PGE_2_, and prostaglandin F2 alpha (PGF_2α_) and TXA2 by their respective synthases. These four primary PGs are spatially and temporally expressed within hair follicles, potentially playing a role in hair loss [10,11,12,15]. 

Many studies suggest that PGE_2_ has been highlighted for its significant contribution to tissue regeneration by promoting the activation of adult stem cells across various injured organs [16,17,18,19,20]. In addition, PGE_2_ was reported to mitigate radiation-induced hair loss in mice [21]. Recent research indicated the involvement of PGF_2α_ in regulating hair growth. Notably, the PGF_2α_ analog, bimatoprost, is approved by the Food and Drug Administration (FDA) in the US, is commonly employed to promote the growth of human eyelashes, and has exhibited effectiveness in cultured human hair follicles [22].

15-PGDH is an enzyme that regulates the intracellular concentrations of PGE_2_ by catalyzing the oxidation of the 15(S)-hydroxyl group on PGE_2_ to its corresponding keto group [23]. Recent research demonstrated that 15-PGDH inhibitors enhance tissue repair by augmentation of PGE_2_ levels [24,25,26,27]. 

Thus, we found DPP, an inhibitor against 15-PGDH, and examined whether DPP ameliorated DHT-damaged HFDPCs and was a potential therapeutic agent for improving hair growth. 

## 2. Results

### 2.1. The Binding Modeling of the Interaction between DPP and 15-PGDH

**Figure 1:**
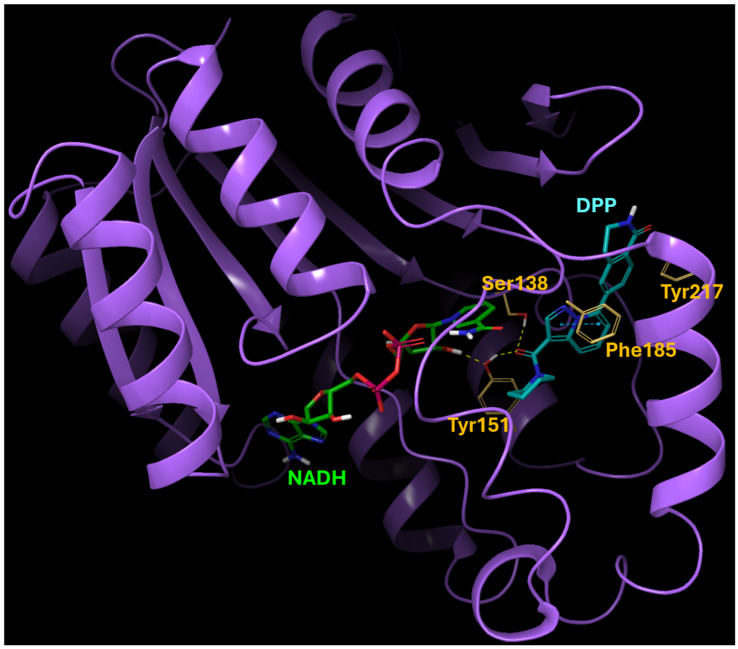
Predicted binding mode of 15-PGDH and DPP complex. The binding is primarily driven by hydrogen-bond interactions with Ser138 and Tyr151, supplemented by a pi-pi interaction involving Phe185. Hydrophobic interactions with Tyr217 also contribute to enhancing the overall binding affinity. The yellow color indicates key residues in the binding site of 15-PGDH.

We originally identified 15-PGDH inhibitor hits by screening a selected set of compounds at Korea Chemical Bank (http://www.chembank.org (accessed on 7 July 2021)) and then discovered DPP by optimizing the lead structure. This was guided by protein-ligand docking with GNINA, a molecular docking software equipped with built-in capabilities to score and refine ligands, leveraging convolutional neural networks. The predicted binding mode of the 15-PGDH and DPP complex is depicted in Figure 1. The binding is predominantly driven by hydrogen-bonding interactions with Ser138 and Tyr151, with additional contribution from the pi-pi interaction involving Phe185. Hydrophobic interactions with Tyr217 also play a role in enhancing the overall binding affinity.

### 2.2. DPP Enhanced the Migration of DHT-Damaged HFDPCs

**Figure 2:**
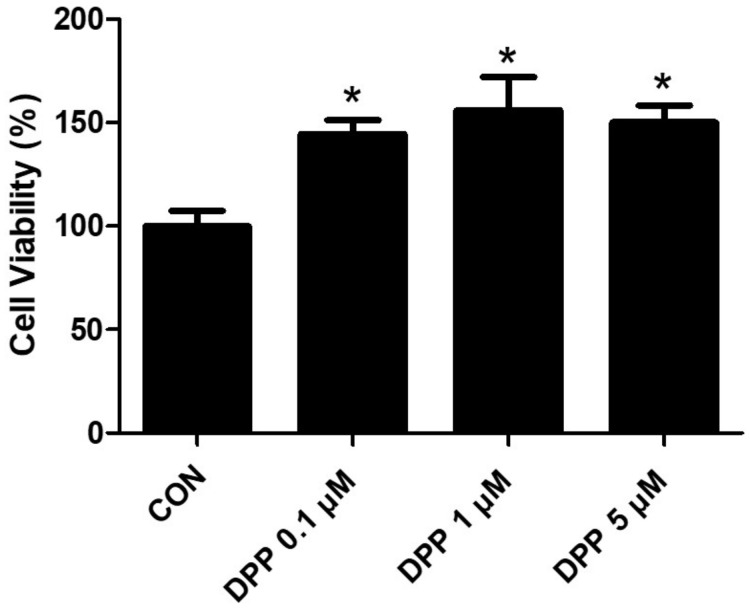
The cell viability of DPP in HFDPCs. The MTT assay of DPP. Cell viability was calculated as the percentage (%) of viable cells relative to untreated cells. All data are expressed as mean ± SD (*n* = 4). * *p* < 0.05 is compared with the control group.

We performed an MTT analysis to examine the potential cytotoxicity of DPP on HFDPCs. As shown in Figure 2, DPP (0.1, 1, 5 μM) showed no cytotoxicity in HFDPCs (Figure 2). In addition, DPP increased cell viability compared to the control group, implying that DPP may promote the cell proliferation of HFDPCs. 

**Figure 3:**
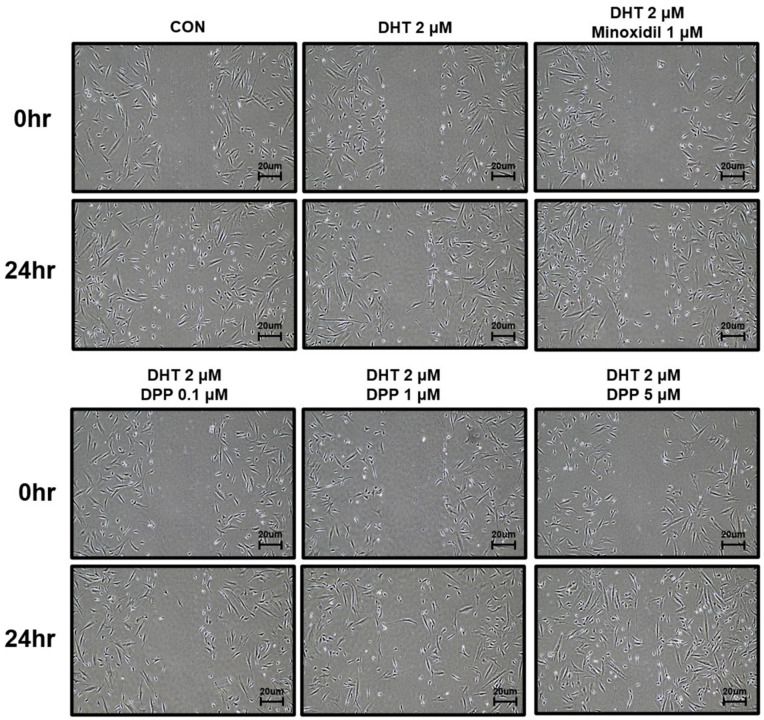
The wound healing effect of DPP in HFDPCs. A wound healing assay was performed on HFDPCs damaged by 1 μM DHT. Each of 0.1, 1, and 5 μM DPP and 1 μM MIX were treated for 24 h, respectively. This wound healing image was taken under a phase contrast microscope. This was a representative image of three independent experiments.

To investigate the wound healing efficacy of DPP in DHT-damaged HFDPCs, a wound healing assay was performed. HFDPCs were scratched in the center of cell culture dishes and treated with 2 μM DHT, DPP (0.1, 1, 5 μM), and 1 μM minoxidil (MIX) as a positive control, respectively. After 24 h, cell growth inhibition was observed in the DHT-treated group, whereas the increase in cell migration significantly occurred in the DHT-treated group added with MIX. Interestingly, we found that DPP significantly enhanced the wound healing efficiency of DHT-damaged HFDPCs in a concentration-dependent manner (Figure 3). 

### 2.3. DPP Increased the Expression Level of Alkaline Phosphatase in DHT-Damaged HFDPCs

**Figure 4:**
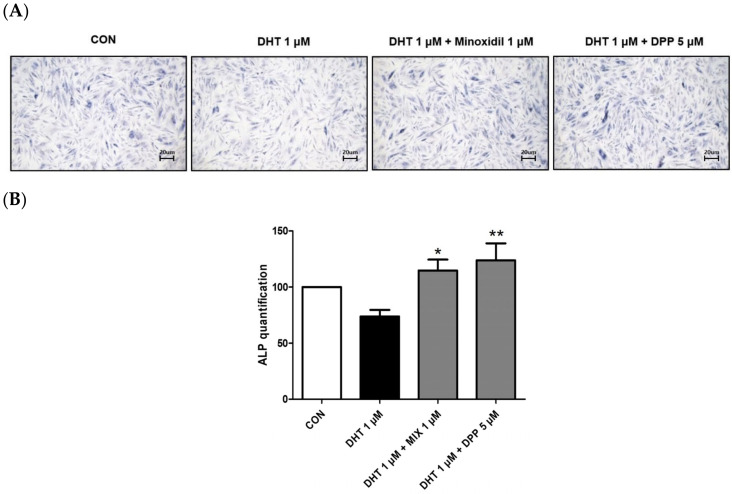
The ALP assay of HFDPCs stimulated by DHT. The ALP assay was conducted on HFDPCs damaged by 1 μM DHT. Each 5 μM DPP and 1 μM MIX were treated for 24 h, respectively. (**A**) This ALP image was taken under a phase contrast microscope. This was a representative image of three independent experiments. (**B**) A graph of the picture using image-J. All data are expressed as mean ± SD (*n* = 3). * *p* < 0.05, ** *p* < 0.01 compared with DHT-treated group.

Alkaline phosphatase (ALP) is essential for cellular physiological processes and tissue regeneration. Its activity is mainly observed in cells with elevated metabolic rates or in tissues undergoing remodeling [28]. Hair growth is significantly linked to alkaline phosphatase activity [29]. This enzyme is crucial for initiating the transition of hair follicles from the telogen to the anagen phase, thus promoting the hair follicle cycle [30]. As expected, treatment with 1 μM MIX enhanced the expression level of ALP in DHT-damaged HFDPCs. We observed that 5 μM DPP treatment significantly increased the expression level of ALP in DHT-damaged HFDPCs compared to the only DHT-treated group (Figure 4). 

### 2.4. DPP Reduced the Reactive Oxygen Level in DHT-Damaged HFDPCs

**Figure 5:**
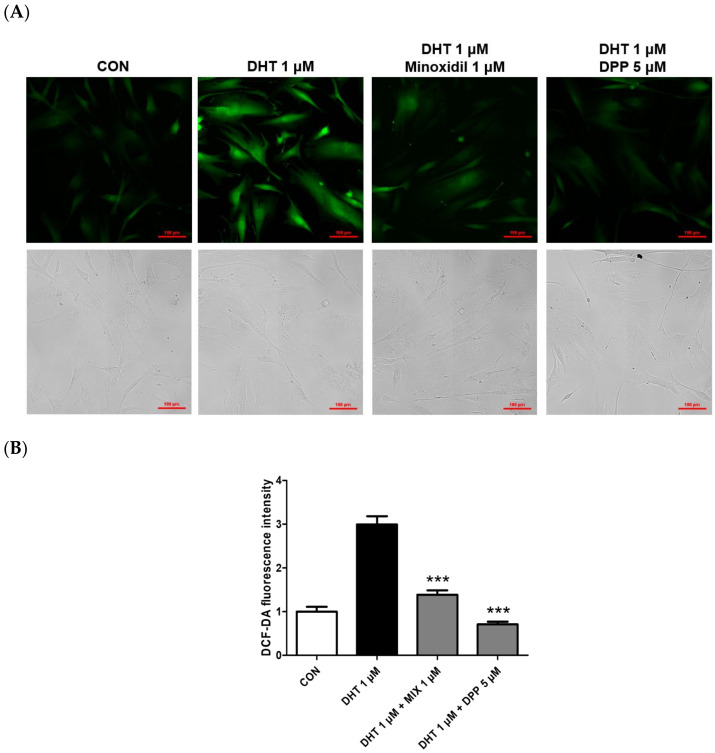
Effects of DPP on ROS levels in DHT-damaged HFDPCs. The DCF-DA ROS assay was conducted on HFDPCs damaged by DHT at 1 μM. 5 μM DPP and 1 μM MIX was pretreated for 24 h followed by DHT damage. (**A**) DCF-DA images were captured using a fluorescence microscope, and the intensity of the green fluorescence represents the ROS concentration. This was a representative image of three independent experiments. (**B**) A graph of the picture using image-J. All data are expressed as mean ± SD (*n* = 3). *** *p* < 0.001 compared with the DHT-treated group.

Depending on internal or external stimuli like ultraviolet radiation and particulate matter, reactive oxygen species (ROS) are chemical compounds generated within cells, and their excessive accumulation leads to cellular damage and various diseases [31,32]. Exposure of HFDPCs to ROS degrades proteins and DNA in hair follicles, triggering inflammation of adjacent tissues. These mechanisms can disrupt hair growth and ultimately result in hair loss [33,34]. A previous report revealed that DHT caused the generation of intracellular ROS in HFDPCs [35]. To measure the levels of ROS within HFDPCs, the DCF-DA assay is performed. As expected, we also found that the ROS level was increased in the only DHT-treated group, whereas the ROS level was lower in the DHT-treated group added with MIX. We also observed that 5 μM DPP significantly downregulated ROS levels generated in DHT-damaged HFDPCs, similar to the control group, meaning that DPP has antioxidant properties (Figure 5).

### 2.5. DPP Recovered the Mitochondrial Potential in DHT-Damaged HFDPCs

**Figure 6:**
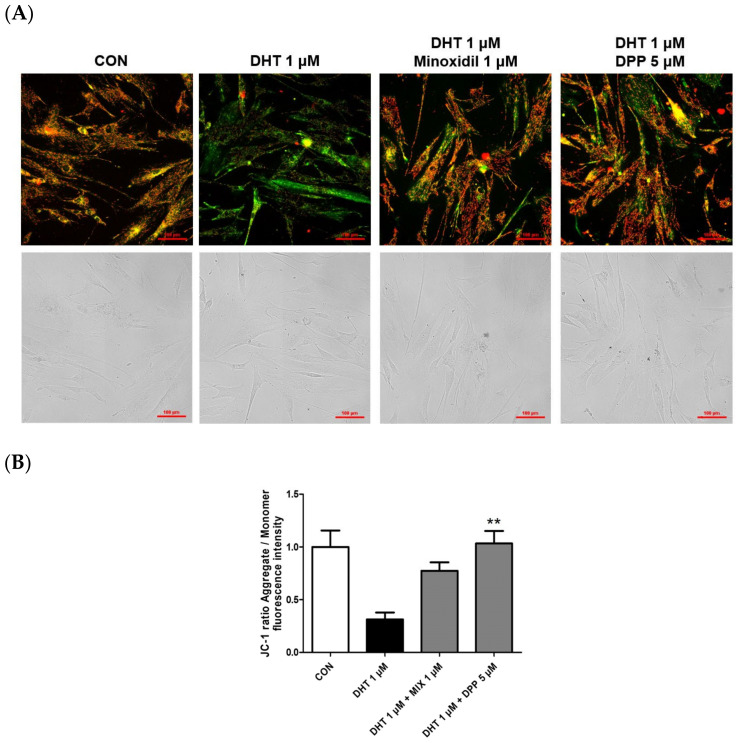
Effects of DPP on mitochondrial membrane potential in DHT-damaged HFDPCs. The JC-1 assay was conducted on HFDPCs damaged by DHT at 1 μM. 5 μM DPP and 1 μM MIX was treated for 24 h. (**A**) JC-1 images were captured using fluorescence microscope. Red puncta, hyperpolarized mitochondria; green puncta, depolarized mitochondria. This was a representative image of three independent experiments. (**B**) A graph of the picture using image-J. All data are expressed as mean ± SD (*n* = 3). ** *p* < 0.01 compared with the DHT-treated group.

Mitochondria play a pivotal role in cellular energy production, and dysfunction in mitochondrial function is linked to cellular impairment [36,37,38]. The JC-1 assay assesses changes in mitochondrial membrane potential, offering an indirect measurement of mitochondrial function [39]. The accumulation of JC-1 dye within mitochondria, dependent on membrane potential, emits green fluorescence around 529 nm for its monomeric state, transitioning to red fluorescence at 590 nm due to the formation of red fluorescent J-aggregates. While a damaged membrane potential is indicated by green fluorescence, a healthy mitochondrial membrane potential is indicated by red fluorescence. We performed a JC-1 assay to evaluate changes in the mitochondrial membrane potential in DHT-damaged HFDPCs treated with DPP. As shown in Figure 6, DHT exhibited green fluorescence compared to the control group. Interestingly, treatment with 5 μM DPP showed red fluorescence in DHT-damaged HFDPCs, implying that DPP restored mitochondrial potential in DHT-damaged HFDPCs. 

### 2.6. DPP Decreased the mRNA Expression Level of Dickkopf-Related Protein 1 (DKK-1), and Interleukin 6 (IL-6) in DHT-Damaged HFDPCs

**Figure 7:**
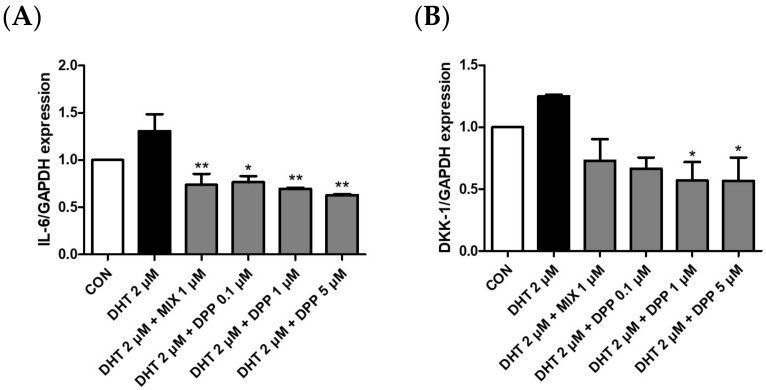
Effects of DPP on mRNA expression levels of IL-6, and DKK-1 in DHT-damaged HFDPCs. Expression levels of mRNA (**A**) IL-6 and (**B**) DKK-1 were determined by qRT-PCR. Each sample was treated with 2 μM DHT, DPP (0.1, 1, 5 μM), and 1 μM MIX, respectively for 5 h (*n* = 3). * *p* < 0.05, and ** *p* < 0.001 is compared with the control group.

To verify the expression of IL-6 and DKK-1 in DHT-damaged HFDPCs, we conducted realtime qRT-PCR analysis. DKK-1 is an antagonist of the Wnt signaling pathway, which is vital for hair growth. Downregulation of DKK-1 expression promotes hair survival and proliferation, whereas its increased expression is strongly correlated with hair growth impairment and the initiation of apoptosis [40,41]. Inflammation induced by IL-6 affects hair growth. Decreasing IL-6 expression can alleviate the effects of stress and inflammation on hair follicles, consequently promoting hair growth [42]. As expected, the mRNA expression levels of DKK-1 and IL-6 were upregulated in the DHT-treated group. We demonstrated that DPP significantly reduced the expression levels of DKK-1 and IL-6 in the DHT-damaged HDFPCs (Figure 7). Therefore, we assumed that the DPP increased the expression of factors beneficial for hair growth, promoted Wnt signaling, and exhibited anti-inflammatory effects.

### 2.7. DPP Increased the Phosphorylation Levels of ERK, AKT β-Catenin, and GSK-3β in DHT-Damaged HFDPCs

The ERK and AKT signaling pathways are key regulators of cell proliferation [43,44]. The ERK signaling pathway plays a crucial role in cell proliferation, whereas AKT is indispensable for transmitting survival signals [44,45]. The Wnt signaling pathway is essential for hair formation and the hair cycle [46,47]. GSK-3β serves as a crucial kinase for regulating catenin stability. β-catenin contributes to cell cycle regulation and activation of genes involved in hair formation, playing a crucial role in ensuring the healthy growth and maintenance of hair [46,47,48]. 

**Figure 8:**
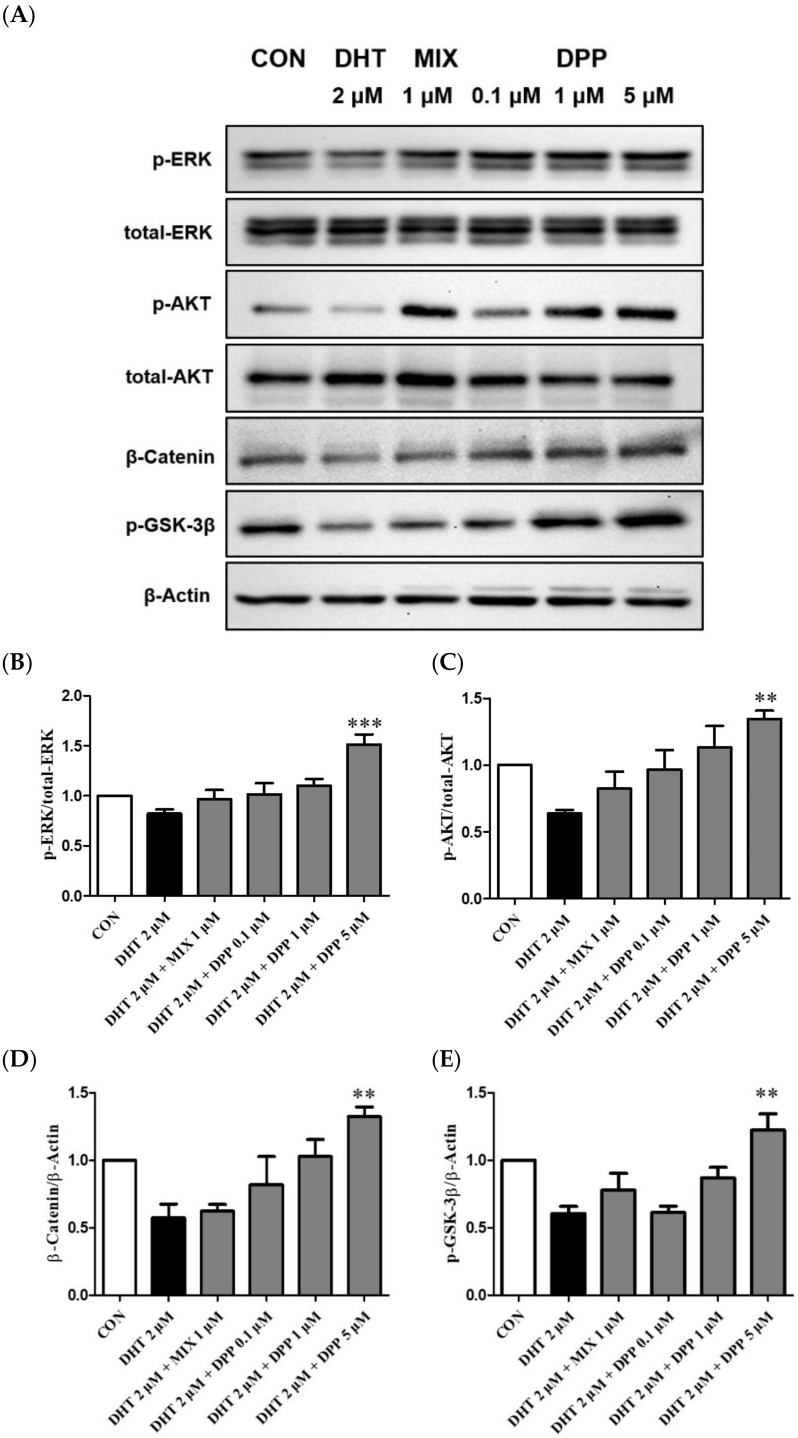
Effect of DPP on the phosphorylation level of ERK, AKT, GSK-3β, and Catenin in DHT-damaged HFDPCs. (**A**) Relative expression of each protein. (**B**) ERK, (**C**) AKT (**D**) β-Catenin, and (**E**) GSK-3β relative expression bar graph of DPP. Each sample was treated with 2 μM DHT, DPP (0.1, 1, 5 μM), and 1 μM MIX for 24 h and then the expression of proteins was analyzed using western blotting. All data are expressed as mean ± SD (*n* = 3). ** *p* < 0.01, *** *p* < 0.001 compared with DHT-treated group.

To explore the underlying mechanisms of DPP, the effects on phosphorylation ERK, AKT, GSK-3β, and β-catenin were evaluated using Western blot analysis. As expected, the phosphorylation levels of p-ERK, p-AKT, GSK-3β, and the expression level of β-catenin were reduced by DHT-damaged HFDPCs. However, DPP treatment enhanced the phosphorylation levels of p-ERK, p-AKT, GSK-3β, and the expression level of β-catenin in DHT-damaged HFDPCs. Especially, DPP exhibited a significant reduction at a concentration of 5 μM (Figure 8). We suggested that DPP promoted cell proliferation through the activation of AKT/ERK /Wnt signaling pathways in DHT-damaged HFDPCs.

### 2.8. DPP Increased the Size of the 3-Dimensional (3D) Spheroid in DHT-Damaged HFDPCs

**Figure 9:**
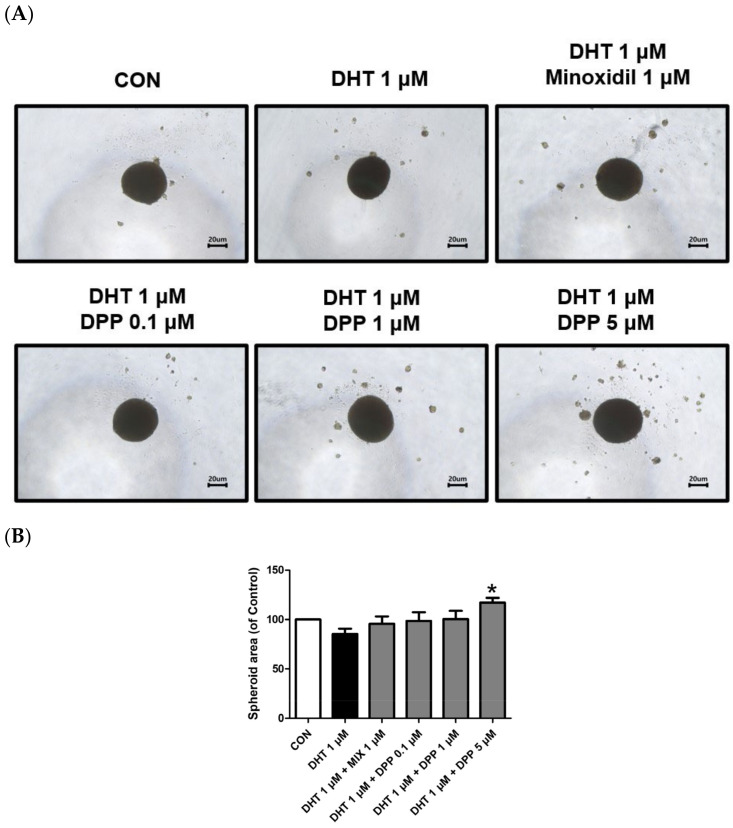
Effects of DPP on the formation of 3D spheroid in DHT-damaged HFDPCs. Treatments with 1 μM DHT, DPP (0.1, 1, 5 μM), and 1 μM MIX were administered at 2-day intervals, and images were taken after 21 days of culture. (**A**) Each image of a 3D spheroid was taken under a phase contrast microscope. This was a representative image of three independent experiments. (**B**) A graph of the picture using image-J. All data are expressed as mean ± SD (*n* = 3). * *p* < 0.05 compared with the DHT-treated group.

HFDPCs possess self-renewal properties like stem cells, enabling three-dimensional culture [49]. A three-dimensional spheroid is utilized due to the three-dimensional interaction and tissue formation capability of cells [50,51]. This model allows us to understand complex cell–cell interactions compared to 2D cell cultures. Interestingly, we demonstrated that 5 μM DPP significantly increased the spheroid size in DHT-damaged HFDPCs compared to the only DHT-treated group (Figure 9), exhibiting a correlation with the improved wound healing efficiency shown in Figure 2.

### 2.9. DPP Stimulated Hair Growth Ex Vivo

**Figure 10:**
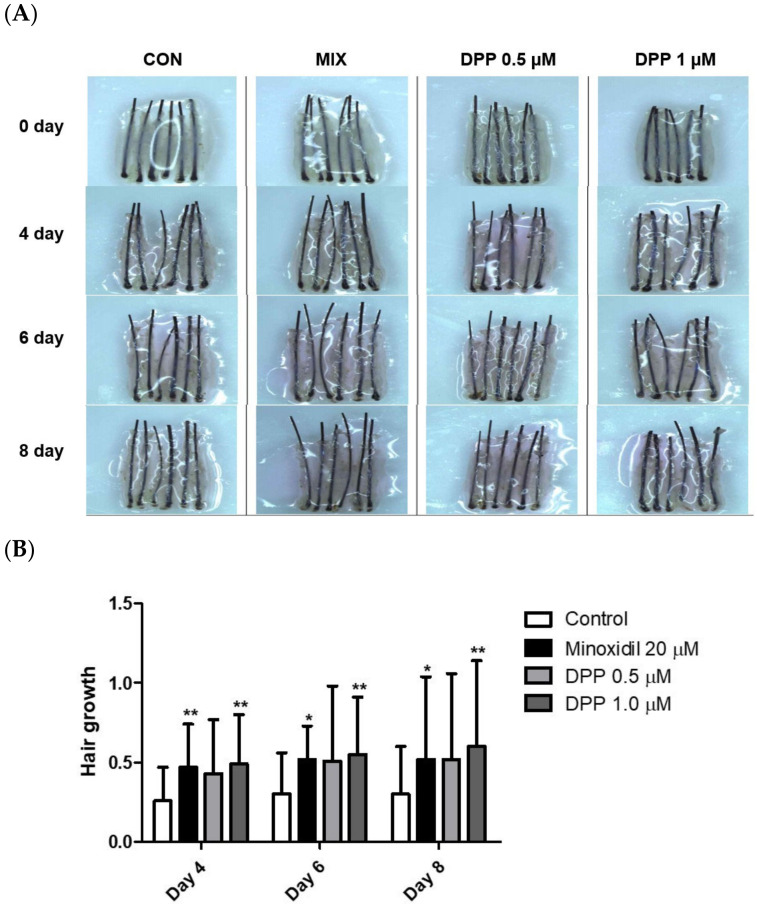
Effects of DPP on hair growth in human hair follicle organ culture. To evaluate the effect of DPP, the anagen human hair follicles were prepared and cultured for 8 days. DPP was treated at concentrations of 0.5 and 1 µM. (**A**) The cultured hair follicles were photo-documented on 4, 6, and 8 days. (**B**) The hair shaft growth was analyzed. MIX was used as a positive control. The data represent the mean ± SD of eighteen follicles. *p*-values were obtained using the Mann–Whitney U test. Significantly different compared with control group (* *p* < 0.05, ** *p* < 0.01).

The hair follicle organ culture model introduced an exceptionally accessible method to evaluate the three-dimensional interactions among epithelial, mesenchymal, and neuroectodermal cells [52]. Additionally, this assay system enables the assessment of how various natural and pharmacological agents influence the growth modulation of complex tissues. As a final step, we investigated whether DPP enhanced human hair growth in an ex vivo human hair follicle organ culture model. As expected, MIX exhibited remarkable hair growth compared to the control group. Interestingly, DPP significantly increased hair growth compared with non-treated control (Figure 10). On day 8 of culture, DPP increased the length of the hair shaft by 10.7% (0.5 µM) and 13.1% (1 µM) compared with non-treated control. 

## 3. Discussion

As the demand for hair loss treatments targeting hair loss increases among numerous patients, the hair loss solution market is also expanding [53]. AGA often causes social and psychological problems for individuals, leading to hair loss due to decreased anagen and increased telogen phases [54]. Considering the side effects of finasteride and MIX, commonly used for AGA and approved by the FDA [55], the development of safer drugs is needed to improve hair loss. 

DHT, a testosterone derivative, mainly triggers this AGA. DHT interacts with the androgen receptor in hair follicle cells to create the AR-DHT complex, which undergoes dimerization and nuclear translocation. This complex, along with AR co-activators, binds to DNA sequences. Moreover, the AR-DHT complex promotes the transcription of TGF-β1/-β2, and DKK-1, resulting in hair follicle miniaturization, shortened anagen phase, and eventual follicle regression leading to alopecia [56]. 

Prostaglandins play a role in regulating hair growth and differentiation [57]. PGE_2_ has been shown to stimulate hair growth following depilation. Thus, to enhance the PGE_2_ level in HFDPCs, we decided to develop an inhibitor of 15-PGDH using our AI-based new drug development program. Our study investigated that DPP increased hair growth in DHT-damaged HFDPCs. Treatment with DPP stimulated cell migration in DHT-damaged HFDPCs (Figure 2). Additionally, it enhanced the activity of alkaline phosphatase, a biomarker for hair follicle cells (Figure 3). ROS is one of the major factors causing hair loss. We found that DPP exhibited significant suppression of ROS compared to the DHT-damaged group (Figure 4). Dysfunction in mitochondria leads to disruption in energy metabolism balance, resulting in ROS production [58]. Interestingly, DPP restored mitochondrial function to levels similar to the control group (Figure 5). 

DKK1 is recognized as a secreted protein that acts as an inhibitor of Wnt signaling, exerting a negative regulatory role [59]. Thus, we demonstrated that DPP antagonized the expression level of DKK-1 elevated in DHT-damaged HFDPCs (Figure 6). The activation of the Wnt signaling pathway increases the expression of genes involved in cell cycle progression, thereby enhancing hair follicle formation and regeneration [60]. β-catenin forms a complex with GSK3β, APC, CK1, and Axin in the cytoplasm. Upon Wnt ligands activation (Wnt ON state), the ligands bind to the receptors, leading to β-catenin translocation into the nucleus and inducing the expression of genes related to hair proliferation. We verified that treatment with DPP enhanced the expression level of β-catenin. DPP promoted cell growth by inhibiting β-catenin degradation through ERK and AKT and by facilitating its nuclear translocation, implying that DPP could effectively prevent hair loss (Figure 8).

The 3D spheroid model generally represents the cell–cell interactions and signaling pathways in dermal papilla cells, which typically exhibit stemness characteristics [61]. This model is instrumental in mimicking realistic conditions of cellular interactions, rendering it a crucial tool for investigating hair follicle formation and regeneration. Interestingly, we revealed that DPP exhibited a significant enlargement in spheroid size, implying that DPP can potentially modulate growth in a tissue-mimicking environment (Figure 9). We finally confirmed that DPP increased hair growth in ex vivo hair follicle organ culture (Figure 10). 

In conclusion, we suggest that DPP can enhance hair growth and serve as a promising component for hair loss improvement by activating ERK/AKT/Wnt signaling pathways.

## 4. Materials and Methods

### 4.1. Chemicals and Reagents

DPP was screened from a chemical library in Innovo Therapeutics Inc., Daejeon, Republic of Korea. 2-propanol, dimethyl sulfoxide (DMSO), DHT, and MIX were purchased from Sigma, Sigma Chemical (St. Louis, MO, USA). The cellular ROS Detection Assay Kit, JC-1-Mitochondrial Membrane Potential Assay Kit, and Alkaline Phosphatase Staining Kit (Purple) were purchased from Abcam (Cambridge, UK). Bovine serum albumin (BSA), BCA protein assay kit, lysis and extraction buffer, and lithium dodecyl sulfate (LDS) sample buffer (4X) were purchased from Thermo Fisher Scientific Inc. (Waltham, MA, USA). HFDPCs, follicle dermal papilla cell growth Medium, and DetachKit were purchased from Promo Cell (Heidelberg, Germany). Dulbecco’s phosphate-buffered saline (DPBS), penicillin-streptomycin, and Cell/tissue culture grade water were purchased from Welgene Inc. (Gyeongsangbuk-do, Republic of Korea). Blotting-grade blocker, 10% Tween 20 solutions, and 10X Tris-buffered saline containing 1% Tween-20 (TBS-T) were purchased from Bio-Rad Inc (Hercules, CA, USA). Fetal bovine serum (FBS) was purchased from Capricorn (Ebsdorfergrund, Germany). Polyvinylidene fluoride (PVDF) membranes for Western blotting analysis were purchased from Roche (Mannheim, Germany). Enhanced chemiluminescence (ECL) prime, a Western blotting detection reagent, was purchased from Cytiva (Marlborough, MA, USA). Antibodies recognizing phospho-AKT, total AKT, phospho-ERK, and total ERK were purchased from Cell Signaling Technology (Beverly, MA, USA). Additionally, antibodies phospho-GSK 3 Beta, GSK 3 Beta, and Catenin were purchased from Santa Cruz Biotechnology (Santa Cruz Biotechnology, Dallas, TX, USA). The EZ-cytox cell viability assay kit was purchased from DoGenBio (Seoul, Republic of Korea). 96-well clear round-bottom ultra-low attachment multiple microplates were purchased from Corning (Glendale, AZ, USA). Confocal dishes were purchased from SPL (Gyeonggi-do, Republic of Korea).

### 4.2. Modeling of DPP

We utilized GNINA (version 1.1) for conducting protein–ligand docking, incorporating support for scoring and optimizing ligands through convolutional neural networks (CNN) [62]. The X-ray structure of the 15-PGDH and ligand complex (PDB 8CWL) was obtained from the RCSB Protein Data Bank. The protein was preprocessed using ProDy to eliminate non-protein components like water, cofactors, and ligands [63]. The 3D structure of DPP was formatted in SD using OpenBabel [64]. Subsequently, docking simulation was conducted with GNINA using the prepared protein and DPP. The binding site was specified to be in the vicinity of the reference ligand, which was extracted from the original PDB. Default settings were applied for all other parameters. The resulting binding poses were ranked based on CNNscore, which varies from 0 (poorest) to 1 (optimal). Finally, we selected the most favorable binding mode by considering the CNNscore and manually examining the binding interactions.

### 4.3. Cell Culture

HFDPCs were obtained from PromoCell (Heidelberg, Germany). The cells were cultured in follicle dermal papilla cell growth medium with a supplement pack and 1% penicillin-streptomycin at 37 °C in a 5% CO_2_ incubator. Every 3 days, 80–90% confluence, cells were detached using a detach kit and transferred to a new 75 mm flask.

### 4.4. Cell Viability Assay

Cell viability was assessed by using the EZ-cytox cell base assay kit. HFDPCs were seeded at 2 × 10^4^ cells/well density in a 96-well plate. After incubation at 37 °C in a 5% CO_2_ incubator for 24 h, HFDPCs were treated with DPP (0.1, 1, and 5 μM) for 24 h. After incubation, the culture medium was aspirated from each well. Then, each well was treated with MTT labeling reagent 10 μL with follicle dermal papilla cell growth medium 100 μL was treated in each well in the dark for 1 h at a 37 °C incubator. Absorbance was measured at 450 nm.

### 4.5. Wound Healing Assay

HFDPCs were seeded at 4 × 10^4^ cell density in a 6-well dish. Subsequently, incubated at 37 °C in a 5% CO_2_ incubator for 24 h, the density of cells reached 80%. Then, a pipette tip (200 μL) was used to create a scratch in a horizontal line at the bottom of the dish, crossing through the confluent cells in the middle. After removing the culture medium, the new culture medium was added and treated with 2 μM DHT, DPP (0.1, 1, and 5 μM), and 1 μM MIX, respectively, and incubated for 24 h at 37 °C in a CO_2_ incubator. To compare images 0 h and 24 h, all dishes are photographed using a microscope. 

### 4.6. Alkaline Phosphatase Staining (ALP) Assay

HFDPCs were plated in a 24-well plate at a density of 3.125 × 10^4^ cells/well and incubated for 24 h. Subsequently, the cells were treated with 1 μM DHT, 5 μM DPP, and 1 μM MIX, respectively, for 24 h at 37 °C in a CO_2_ incubator. After treatment, the cells were washed with PBS-T and fixed with a fixing solution for 2 min. The cells were stained with an alkaline phosphatase staining solution for 24 h. And then it was washed with DPBS. The purple-stained colonies were counted and compared to the colorless colonies using a light Nikon microscope (Tokyo, Japan).

### 4.7. DCF-DA ROS Assay

HFDPCs were seeded in a confocal dish at 2.5 × 10^4^ cells/mL density and incubated for 24 h. The cells were treated with 1 μM DHT, 5 μM DPP, and 1 μM MIX, respectively, for 24 h at 37 °C in a CO_2_ incubator. Subsequently, the cells were washed with DPBS 2 times and stained with 10 μM 2,7-Dichlorofluoroscin diacetate (DCF-DA) for 15 min. Then, the cells were washed with DPBS and added DPBS 200 μL for the measure. The fluorescence was measured with a fluorescence microscope purchased from Nikon (Tokyo, Japan). 

### 4.8. Measurement of Mitochondrial Membrane Potential

HFDPCs were seeded in a confocal dish at 2.5 × 10^4^ cells/mL density and incubated for 24 h. Then, the cells were treated with DHT 1 μM, 5 μM DPP, and MIX 1 μM, respectively, for 24 h at 37 °C in a CO_2_ incubator. Subsequently, the cells were washed with DPBS 2 times and were stained with 5 μM JC solution for 15 min. The cells were washed with DPBS and added DPBS 200 μL for the measure. The fluorescence was measured with a Nikon fluorescence microscope (Tokyo, Japan). 

### 4.9. Quantitative Real-Time Polymerase Chain Reaction 

HFDPCs were plated in a 6-well dish at a density of 7.0 × 10^4^ cells/mL. Then, the cells were cultured for 24 h at 37 °C in a CO_2_ incubator. Subsequently, the cells were treated with 2 μM DHT, DPP (0.1, 1, and 5 μM), and 1μM MIX after 24 h of incubation. Following this, the cells underwent two washes with DPBS and were utilized for RNA extraction. RNA was purified using TRIzol reagent, and the purified total RNA (4 μg) was synthesized into cDNA using the RevertAid First Strand cDNA synthesis kit. Subsequently, the assays were conducted utilizing TaqMan Universal Master Mix II, with UNG, for quantitative real-time polymerase chain reaction (qRT-PCR). The reaction mixture (total volume 20 μL) comprised 6 μL of DEPC water, 10 μL of TaqMan Universal Master fast Mix II, 3 μL of cDNA, and 1 μL of Assay primers.

### 4.10. Western Blot Analysis

HFDPCs were seeded at density 5 × 10^4^ cells/well in a 100 mm dish and incubated for 24 h at 37 °C in a CO_2_ incubator. The cells were treated with DHT 2 μM, DPP (0.1, 1, and 5 μM), and 1 μM MIX for 24 h. The cells were washed two times with DPBS and lysed using RIPA buffer. Then, the cell lysates were sonicated for 10 min. Total protein concentration was measured using a BCA assay with bovine serum albumin as standard. The protein of 30 μg/μL for phospho-AKT, AKT, phospho-ERK, ERK, phospho-GSK-3β, and β-catenin were separated on a sodium dodecyl sulfate-polyacrylamide gel electrophoresis (SDS-PAGE) and transferred onto polyvinylidene difluoride (PVDF) membranes. The membranes were blocked by 3% non-fat dry milk for 1 h. Primary antibodies against p-AKT, AKT, p-ERK, and ERK were diluted at 1:1000 in a blocking solution. Primary antibodies against p-GSK-3β and β-catenin were diluted at 1:500 in a blocking solution. These primary antibodies were incubated for 1 h at room temperature and then washed 3 times with TBS-T. Horseradish peroxidase-conjugated secondary antibodies were used at a 1:5000 concentration for 1 h at room temperature and then washed 3 times with TBS-T. Finally, the ECL reagent detected the immunoreactive bands, and images were visualized with Invitrogen iBright 1500 (Waltham, MA, USA). The results were analyzed by Fiji Image J (Win 64-bit) software.

### 4.11. Three-Dimensional Spheroid Culture of HDPCs

HFDPCs were seeded in 96-well clear round-bottom ultra-low attachment multiple microplates at a density of 5 × 10^4^ cells/well and incubated for 24 h. Subsequently, the cells were treated with 1 μM DHT, DPP (0.1, 1, and 5 μM), and 1 μM MIX, respectively, at 37 °C in a CO_2_ incubator. The diameter of the spheroids was measured using a light Nikon microscope (Nikon, Japan).

### 4.12. Human Hair Follicle Organ Culture

Human scalp skin was obtained from nonbalding areas of patients undergoing hair transplant surgery with written consent and approval by the Institutional Review Board of Dankook University Hospital (DKUH 2021-12-005). Human hair follicles were isolated by microdissection under the microscope. Anagen VI hair follicles were chosen for the study. Each treatment group consisted of 6 hair follicles, and the experiments were repeated 3 times. Isolated hair follicles were maintained in William’s E medium supplemented with 10 µg/mL insulin, 10 ng/mL hydrocortisone, 2 mM L-glutamine, and 10 U/mL penicillin, 100 ug/mL streptomycin, and 25 µg/mL amphotericin B. All cultures were incubated at 37 °C in an atmosphere of 5% CO_2_ and 95% air.

### 4.13. Statistical Analysis

All results are expressed as the mean ± standard deviation (SD) of three independent experiments. According to Tukey’s Multiple Comparison Test, all statistical analyses were performed using GraphPad Prism 5.0 software (San Diego, CA, USA) through a one-way analysis of variance (ANOVA). Statistical significance between the groups was accepted for *p* values < 0.05.

## Data Availability

The data presented in this study are available on request from the corresponding author.
